# Differences in postpartum depressive symptoms across survey waves among Chinese mothers and their association with structural resources: a repeated cross-sectional study based on CFPS data

**DOI:** 10.3389/fmed.2026.1865725

**Published:** 2026-07-02

**Authors:** Pingping Wang, Jin Yao, Yaqiong Li, Zhanjun Zhang, Xiaorong Huang

**Affiliations:** 1 Department of Medical Laboratory, Luoyang Maternal and Child Health Hospital, Luoyang, Henan, China; 2 Department of Infection and Public Health Management, The Second Affiliated Hospital of Henan University of Science and Technology, Luoyang, Henan, China; 3 Medical Imaging Department, Luoyang Maternal and Child Health Hospital, Luoyang, Henan, China; 4 Luoyang Research Center for Inheritance and Innovation of Chinese Historical Civilization, Luoyang Institute of Science and Technology School of Marxism (LIT), Luoyang, Henan, China

**Keywords:** China family panel studies, maternal mental health, postpartum depression, repeated cross-sectional study, structural resources

## Abstract

**Objective:**

Using China Family Panel Studies data (CFPS; 2016, 2018, 2020, and 2022), this study examined between-sample differences in postpartum depressive symptoms across survey waves in China and their association with structural resources.

**Methods:**

This repeated cross-sectional study included 827 mothers living in households with 0-year-old infants, identified by linking child questionnaires, family relationship files, and adult questionnaires. Depressive symptoms were measured using the CES-D total score. Elevated depressive symptoms were defined as CES-D ≥ 36, approximately corresponding to the conventional CES-D ≥ 16 cutoff. Structural resources were measured using an index based on educational attainment, employment status, and urban–rural residence. Descriptive statistics, multiple linear regression, binary logistic regression, and sensitivity analyses were conducted.

**Results:**

Mean CES-D scores across the four survey-wave samples were 32.1 ± 7.0, 32.8 ± 6.3, 32.8 ± 7.7, and 34.2 ± 8.4. The proportions with elevated depressive symptoms were 25.3, 28.5, 27.4, and 31.4%. In fully adjusted models, the 2022 sample had higher CES-D scores (*β* = 3.02, SE = 0.99, *p* = 0.002) and higher odds of elevated depressive symptoms (OR = 2.56, 95% CI: 1.33–4.92, *p* = 0.005) than the 2016 sample. The structural resource index was negatively associated with elevated depressive symptoms (OR = 0.88, 95% CI: 0.77–1.00, *p* = 0.046), but this association was attenuated under a stricter threshold.

**Conclusion:**

Postpartum depressive symptoms differed across CFPS survey-wave samples. Higher structural resources were modestly associated with lower odds of elevated depressive symptoms.

## Introduction

Postpartum depression (PPD) is one of the most common mental health problems experienced by women after childbirth. It not only substantially affects mothers’ emotional well-being, social functioning, and quality of life, but may also have long-term adverse effects on children’s cognitive, emotional, and social development through mother–infant interaction, parenting practices, and family relationships (1–3). Several systematic reviews have identified PPD as an important global public health concern, with marked variations in prevalence across countries and social groups (4, 5). In China, as childbirth-related policies continue to change, family caregiving needs evolve, and women’s family and work role pressures receive increasing attention, the identification, prevention, and intervention of postpartum depression have gradually become important issues in public health, maternal and child health, and social work.

In recent years, with the development of social epidemiology and life-course research, scholarly attention has gradually shifted from narrow individual psychological or biological explanations toward understanding postpartum depression within broader social structures and family contexts (6, 7). A substantial body of research has shown that structural socioeconomic factors, such as educational attainment, employment status, household economic conditions, and urban–rural disparities, are closely associated with women’s risk of perinatal and postpartum depression (8–10). At the same time, support within the family, such as partner relationships and intergenerational support, as well as wider social support networks, may play an important buffering role between social structure and individual mental health (11–13). This shift in perspective suggests that postpartum depression is not merely an individual emotional disorder; rather, its onset and development are deeply embedded in the resource conditions, family division of labor, and social support environments in which women live.

Existing studies have generated considerable evidence on the relationship between family support and postpartum depression. Some studies have emphasized the protective role of partner support, marital quality, and family functioning in reducing the risk of postpartum depression (14–16), whereas others have focused on intergenerational childcare support, particularly changes in maternal mental health in families where grandparents participate in infant care (17, 18). Nevertheless, this line of research still has three important limitations that warrant further attention.

First, much of the existing literature is based on single-region or specific-population samples and lacks both national representativeness and a temporal dimension. Most related studies rely on hospital-based, community-based, or urban samples, particularly in economically developed regions (14, 17, 19). Although such studies are valuable for capturing the micro-level mechanisms of family support, they remain limited in explaining differences in postpartum depression risk across social groups and in examining the dynamics of postpartum depressive symptoms under broader social structural changes. In addition, most existing studies are based on single-time-point data, and nationally representative evidence remains limited regarding whether maternal postpartum mental health differs across survey waves.

Second, socioeconomic status and family support are often treated as separate domains, making it difficult to reveal the pathways through which structural inequality operates. In many studies, socioeconomic status (SES) is included merely as a control variable, whereas family support is analyzed as an independent psychosocial factor (15, 16). Although this approach simplifies statistical modeling, it may theoretically underestimate the ways in which structural resources shape the forms, quality, and mental health implications of family support (6, 10). In the context of social transformation, educational expansion, increasing female labor force participation, and changes in urban–rural resource disparities may further affect patterns of support within families and their mental health implications. Therefore, treating socioeconomic status only as a background variable may be insufficient for revealing its deeper role in postpartum depression risk.

Third, existing research on “support” has tended to emphasize subjective perceptions, while paying less attention to distinguishing structural resource conditions reflected by objective social position from support subjectively experienced by individuals. Many studies have relied on subjective scales of social support or family functioning (14, 20), but relatively few have constructed comparable indicators of structural resources based on objective social position and living conditions. Such measures are useful for identifying individuals’ perceived experiences of support, yet they remain limited in explaining how differences in resource distribution shape maternal mental health. This also constrains, to some extent, the interpretive value of related research for public policy and social intervention.

To address these limitations, the present study introduces the perspective of structural resources, emphasizing how mothers’ structural resource conditions in terms of educational attainment, employment status, and urban–rural residence are associated with postpartum depression risk. Specifically, this study regards education, employment, and urban–rural residence as important dimensions reflecting mothers’ access to social resources and relative social position, and uses them to construct a composite structural resource indicator. Unlike prior studies that focus primarily on whether family members provide help, this study is concerned with whether mothers possess the concrete conditions needed to access and mobilize resources within both family and society, and to share the pressures of childcare and daily life.

Using four waves of nationally representative data from the China Family Panel Studies (CFPS), spanning 2016 to 2022, this study examines: (1) overall differences in depressive symptoms among postpartum mothers across survey waves; (2) the associations of structural resources with both the level of postpartum depressive symptoms and the risk of elevated depressive symptoms; and (3) whether structural resources remain associated with postpartum depression risk after adjusting for relevant demographic and health-related factors.

By situating postpartum depression within the intersecting social context of structural health inequality and family support, this study seeks to advance understanding of the social determinants of postpartum depression from the perspective of structural resources, including education, employment, and urban–rural resource conditions. It also aims to provide empirical evidence for maternal and child mental health policy, community support services, and related social work interventions.

### Research hypotheses and study contributions

Building on the above literature review and the limitations identified in prior research, this study proposes the following hypotheses.

*H1*: Levels of depressive symptoms and the proportion of elevated depressive symptoms among postpartum mothers may differ across CFPS survey-wave samples from 2016 to 2022.

*H2*: Higher levels of structural resources are associated with relatively lower levels of postpartum depressive symptoms and a lower likelihood of belonging to the elevated depressive symptoms group.

*H3*: Compared with individual socioeconomic indicators, the composite structural resource index may show a relatively more stable association with elevated depressive symptoms among postpartum mothers.

It should be noted that these hypotheses are mainly derived from existing literature and theoretical reasoning. They are intended to examine possible associations between different social resource conditions and postpartum mental health, rather than to assume a strict causal direction. Given the repeated cross-sectional design of this study, the findings are more appropriately interpreted as comparisons across different survey-wave samples rather than as evidence of longitudinal within-person change.

On this basis, the contributions of this study are as follows.

First, using nationally representative repeated cross-sectional data, this study analyzes differences in depressive symptoms among postpartum mothers across survey waves from 2016 to 2022, thereby providing additional evidence for understanding the population-level characteristics of postpartum maternal mental health in China.

Second, this study adopts a structural resource perspective rather than relying solely on subjective perceptions of support. By incorporating educational attainment, employment status, and urban–rural residence into the same analytical framework, it reconsiders the social determinants of postpartum depression.

Third, this study further distinguishes structural resource conditions from family caregiving support as two different levels of supportive factors, thereby providing a new basis for discussing the possible roles of family support and social structural factors in postpartum mental health.

Fourth, the findings may provide empirical implications for maternal and child mental health policy, community support services, and medical social work practice, particularly in identifying structurally vulnerable groups and improving community-based support systems.

## Methods

### Data source and study population

This study used data from four waves of the China Family Panel Studies (CFPS), conducted in 2016, 2018, 2020, and 2022. The CFPS is a nationally representative longitudinal survey administered by the Institute of Social Science Survey at Peking University. It adopts a multistage, stratified, probability-proportional-to-size sampling design and covers multiple provinces in mainland China. The survey collects information on socioeconomic conditions, health, and family relationships at the individual, household, and community levels, and has been widely used in research on population health and social inequality in China.

This study employed a repeated cross-sectional design to compare depressive symptoms and related factors among postpartum mother samples across different survey waves. Because the participants included in each wave were not the same individuals, the findings mainly reflect overall differences between samples from different survey years and should not be interpreted as longitudinal trajectories of the same population over time.

The target population of this study was mothers living in households with a 0-year-old infant in each of the four survey waves. Because the CFPS was not specifically designed for postpartum populations, the analytic sample was constructed by first identifying 0-year-old infants from the child questionnaire and then linking them to their biological mothers using family relationship files together with information from the adult questionnaire. It should be noted that the CFPS records children’s age but does not provide exact delivery dates. Therefore, “postpartum mothers” in this study refers operationally to women living in households with 0-year-old infants, which broadly corresponds to mothers within the first postpartum year, but does not allow further distinction by exact postpartum month or postpartum stage.

### Sample construction and inclusion criteria

In each survey wave, infants aged 0 years were first identified from the child questionnaire. Their biological mothers were then matched using family relationship files or household member identification variables, after which maternal information from the adult questionnaire was merged into the analytic dataset. Cases were included in the analysis if they met the following criteria:

(1) There was a 0-year-old infant in the household;

(2) The biological mother could be successfully identified and matched; and

(3) The mother had valid information on the CES-D depression scale and the core explanatory variables.

For missing data handling, this study adopted a complete-case analysis strategy. In the regression models, only cases with complete information on the CES-D outcome variable, structural resource indicators, and main covariates were included. Because some variables had missing values, particularly household income, which had a relatively high proportion of missingness, income was not included in the main models.

A total of 827 mothers were ultimately included in the analytic sample, including 297 in 2016, 263 in 2018, 146 in 2020, and 121 in 2022. The final sample size in each wave was determined not only by the number of 0-year-old infants captured in that wave, but also by the success of maternal identification, family relationship matching, and the completeness of key variables. As a result, the sample sizes in the later waves were relatively smaller. After further applying complete-case criteria, the effective sample size for the fully adjusted models was 588.

**Figure 1:**
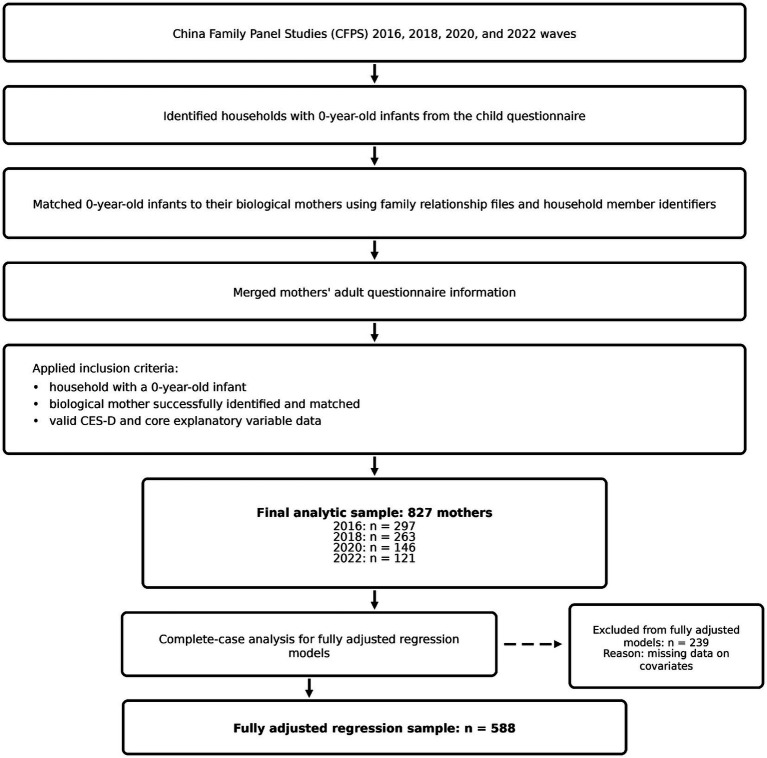
Flowchart of sample selection and analytic samples. Postpartum mothers were operationally defined as women living in households with 0-year-old infants. Because CFPS records child age but not exact delivery date, exact postpartum month could not be determined.

The sample selection and inclusion process is shown in Figure 1.

## Measures

### *Outcome* var*iables*

In this study, the total CES-D score was used to measure depressive symptoms. The CES-D scale used in the CFPS adopts a 20–80 scoring scheme, which differs from the conventional CES-D 0–60 scoring system. Therefore, both a continuous outcome and a binary outcome were used to improve the robustness and comparability of the findings.

First, the total CES-D score was treated as a continuous variable to reflect the overall level of maternal depressive symptoms.

Second, the elevated depressive symptoms group was defined using the 75th percentile of the CES-D distribution in the pooled sample across the four survey waves, which corresponded to approximately 36 points. This binary outcome was used to identify a relatively high-risk subgroup within the sample and should not be interpreted as a clinical diagnosis of postpartum depression.

In addition, to examine the robustness of the findings, a sensitivity analysis was conducted using CES-D ≥ 40 as a stricter threshold, which approximately corresponds to the conventional CES-D ≥ 20 criterion.

### Core explanatory variable: structural resource index

The core explanatory variable in this study was the structural resource index, which was designed to comprehensively capture mothers’ resource position in terms of socioeconomic and living conditions. The concept of “structural resources” was adopted because education, employment, and urban–rural residence not only reflect individual-level socioeconomic characteristics, but also indicate, to some extent, mothers’ relative position in accessing social opportunities, public resources, and social support.

The index consisted of three indicators: educational attainment, employment status, and urban–rural residence. Educational attainment mainly reflects human capital and the accumulation of long-term social opportunities. Employment status reflects labor market participation and economic independence. Urban–rural residence, in the Chinese context, reflects structural differences in access to public services, social welfare, and other resources.

Because these three variables were measured on different scales, the education variable was first standardized using a z-score transformation. Employment status was coded as 1 for employed and 0 for not employed, and urban–rural residence was coded as 1 for urban and 0 for rural. The standardized education score was then directly summed with employment status and urban–rural residence to form a continuous structural resource index.

An equal-weighting approach was used because the aim of this study was to construct a composite indicator reflecting mothers’ overall resource conditions, rather than to estimate the independent contribution of each individual dimension. Higher index values indicate more favorable structural resource conditions.

In addition, to examine the robustness of different index construction methods, principal component analysis (PCA) was further used to construct an alternative structural resource index for sensitivity analysis.

### Covariates

Based on previous research on postpartum depression and the availability of variables in the CFPS, maternal age, marital status, self-rated health, and number of children were further included as covariates.

Maternal age was entered into the models as a continuous variable. Marital status was coded as 1 for married and 0 for other statuses. Self-rated health was coded according to the health-related variables in the CFPS. Number of children was included to reflect the level of family childcare burden.

Because the income variable had a relatively high proportion of missing values in the present sample, including it in the models would have substantially reduced the effective sample size and affected the stability of the estimates. Therefore, income was not included in the main analysis models and was instead addressed as a limitation of the study.

## Statistical analysis

This study employed a repeated cross-sectional design. Accordingly, the statistical analysis focused on comparing overall differences in maternal depressive symptoms and related factors across survey years, rather than examining longitudinal within-person changes over time.

First, descriptive statistics were used to summarize the sociodemographic characteristics of the samples from the four survey waves, as well as CES-D scores, the proportion of mothers with elevated depressive symptoms, and the structural resource index. Continuous variables are presented as means and standard deviations, and categorical variables are presented as frequencies and percentages.

Second, to systematically assess the associations of survey-wave sample and structural resources with postpartum depressive symptoms, two types of multivariable regression models were estimated, namely a multiple linear regression model and a binary logistic regression model. The multiple linear regression model used the total CES-D score as a continuous dependent variable, whereas the binary logistic regression model used elevated depressive symptoms as the dependent variable.

Survey year was entered as a categorical variable, with 2016 as the reference category. The structural resource index was included as the main continuous explanatory variable. Maternal age, marital status, self-rated health, and number of children were included as covariates in the fully adjusted model. In the linear regression models, regression coefficients (*β*), standard errors (SEs), and *p*-values are reported. In the logistic regression models, odds ratios (ORs) and 95% confidence intervals (CIs) are reported. All statistical tests were two-sided, and a *p*-value of less than 0.05 was considered statistically significant.

In addition, two sensitivity analyses were conducted. First, CES-D ≥ 40 was used as a stricter threshold for elevated depressive symptoms, and the logistic regression analysis was repeated. Second, principal component analysis (PCA) was used to construct an alternative structural resource index, and the results were compared with those of the main analysis to assess robustness.

## Missing data handling

Special missing-value codes in the CFPS data, such as −10 to −1 and 77–79, were uniformly recoded as missing values (NA). The regression analyses were based on a complete-case approach, meaning that only cases with complete information on the core variables and covariates were included. As a result, the effective sample size may vary slightly across models.

It should be noted that the income variable had a relatively high proportion of missing values in the postpartum mother sample. Therefore, it was not included in the main models in order to avoid a substantial reduction in the effective sample size and potential instability in model estimation.

## Statistical software

All data cleaning and statistical analyses were conducted in R software, version 4.2.0 or later. Data management was primarily performed using packages such as dplyr and haven, while regression modeling and result processing were carried out using base statistical functions and related extension packages. Principal component analysis was conducted using built-in statistical functions in R.

## Results

### Sample composition and inclusion

Using data from the 2016, 2018, 2020, and 2022 waves of the CFPS, this study defined the target population as mothers living in households with 0-year-old infants. The analytic sample was constructed by identifying 0-year-old infants from the child questionnaire, matching them to their biological mothers through family relationship files, and then merging the mothers’ adult questionnaire data. A total of 827 mothers were included in the final analytic sample, including 297 in 2016, 263 in 2018, 146 in 2020, and 121 in 2022.

Because some variables had missing values, the regression models were based on a complete-case approach, resulting in further reductions in the effective sample size across models. The effective sample size for the fully adjusted model was 588. The final sample size in each wave was determined not only by the number of 0-year-old infants captured in that wave, but also by the success of maternal identification, family relationship matching, and the completeness of key variables. As a result, the sample sizes in the later waves were relatively smaller.

In addition, because this study used a repeated cross-sectional design, the mothers included in different survey years were not the same individuals. Therefore, differences across years should be interpreted as overall differences between samples from different waves rather than as longitudinal changes in the same population over time. Because the CFPS records only children’s age and does not provide exact delivery dates, mothers in this study were defined as women living in households with 0-year-old infants. This definition broadly corresponds to women within the first postpartum year, but does not allow further distinction by exact postpartum month.

### Descriptive characteristics of maternal depressive symptoms and structural resources across the four waves

As shown in Table 1, the mean age of mothers across the four survey-wave samples was approximately 28–30 years, with slightly higher mean ages observed in the later-wave samples. CES-D scores were generally in the range of 32–34 points, with mean scores of 32.1 ± 7.0 in 2016, 32.8 ± 6.3 in 2018, 32.8 ± 7.7 in 2020, and 34.2 ± 8.4 in 2022. Considering that the CES-D scale used in the CFPS adopts a 20–80 scoring scheme, this study further used CES-D ≥ 36 as the cutoff for elevated depressive symptoms. This threshold approximately corresponds to the conventional CES-D ≥ 16 screening criterion under the 0–60 scoring system. Based on the CES-D ≥ 36 definition, the proportions of mothers with elevated depressive symptoms were 25.3% in 2016, 28.5% in 2018, 27.4% in 2020, and 31.4% in 2022, with the highest proportion observed in 2022.

**Table 1:** Descriptive characteristics of mothers with 0-year-old infants in the CFPS, 2016–2022.

Variable	2016 (*n* = 297)	2018 (*n* = 263)	2020 (*n* = 146)	2022 (*n* **=** 121)	Total (*n* = 827)
Maternal age (years)	27.7 ± 3.7	28.1 ± 3.8	29.3 ± 4.1	29.7 ± 4.3	28.5 ± 4.0
CES-D depressive symptom score	32.1 ± 7.0	32.8 ± 6.3	32.8 ± 7.7	34.2 ± 8.4	32.9 ± 7.3
Median CES-D score (IQR)	32 (26, 36)	32 (28, 36)	32 (28, 37.5)	34 (28, 38)	—
Elevated depressive symptoms† (%)	25.3	28.5	27.4	31.4	27.6
Educational attainment (1–7)	3.5 ± 1.7	3.7 ± 1.6	3.9 ± 1.6	4.1 ± 1.7	3.7 ± 1.7
Employed (%)	41.5	43.8	46.2	47.9	43.5
Urban residence (%)	46.4	55.5	51.4	54.3	51.6
Structural resource index	0.78 ± 1.47	0.94 ± 1.41	1.15 ± 1.58	1.26 ± 1.52	0.97 ± 1.48

Continuous variables are presented as mean ± standard deviation. † Elevated depressive symptoms were defined as a CES-D score ≥ 36, a threshold that approximately corresponds to the conventional CES-D ≥ 16 screening criterion. Educational attainment was coded using the original CFPS categorical education variable, with higher values indicating higher educational levels. The structural resource index was constructed from three standardized indicators: educational attainment, employment status, and urban–rural residence.

Compared with the 2016 and 2018 samples, the 2020 and 2022 samples had relatively higher structural resource index scores: 0.78 ± 1.47 in 2016, 0.94 ± 1.41 in 2018, 1.15 ± 1.58 in 2020, and 1.26 ± 1.52 in 2022. Similar descriptive patterns were also observed for educational attainment, employment rate, and the proportion of urban residence.

In addition, the proportion of mothers reporting poorer self-rated health was slightly higher in the later-wave samples, whereas marital status and number of children showed relatively limited differences across survey-wave samples. Overall, although mothers in the later-wave samples tended to have more favorable structural resource conditions, lower depressive symptom levels were not observed in these samples. Both the mean CES-D score and the proportion of mothers with elevated depressive symptoms were highest in the 2022 sample among the four survey-wave samples.

### Continuous CES-D outcome: associations with survey-wave sample and structural resources

Table 2 presents the results of the multiple linear regression analysis using the total CES-D score as the continuous outcome. After adjusting for maternal age, marital status, self-rated health, and number of children, mothers in the 2022 sample had significantly higher CES-D scores than those in the 2016 sample (*β* = 3.02, SE = 0.99, *p* = 0.002). The differences between 2018 and 2016, and between 2020 and 2016, did not reach statistical significance.

**Table 2:** Multiple linear regression analysis of maternal CES-D depressive symptom scores, 2016–2022.

Variable	*β* (SE)	*p*
Survey year (ref: 2016)		
2018	0.88 (0.71)	0.214
2020	1.14 (0.92)	0.216
2022	3.02 (0.99)	0.002
Structural resource index	−0.27 (0.20)	0.178
Maternal age	−0.15 (0.06)	0.019
Marital status (married = 1)	−0.61 (0.88)	0.488
Self-rated health	2.31 (0.39)	<0.001
Number of children	0.18 (0.15)	0.229
Constant	33.94 (2.17)	<0.001

*n* = 588. *β* indicates the regression coefficient, and SE indicates the standard error. The model adjusted for maternal age, marital status, self-rated health, and number of children. Adjusted *R*^2^ = 0.116.

Maternal age was significantly and negatively associated with CES-D scores (*β* = −0.15, SE = 0.06, *p* = 0.019), suggesting that older mothers tended to report relatively lower depressive symptom scores. Meanwhile, self-rated health was significantly and positively associated with CES-D scores (*β* = 2.31, SE = 0.39, *p* < 0.001), indicating that mothers with poorer health status were more likely to report higher levels of depressive symptoms. Marital status and number of children were not statistically significant in the model.

The structural resource index was negatively associated with continuous CES-D scores (*β* = −0.27, SE = 0.20), but this association did not reach statistical significance in the fully adjusted model (*p* = 0.178). This result suggests that there may be some association between structural resources and the overall level of maternal depressive symptoms, but the linear effect was relatively limited.

Compared with the initial model adjusted only for maternal age, the explanatory power of the fully adjusted model improved substantially, with an adjusted *R*^2^ of 0.116. This suggests that postpartum depressive symptoms may be jointly influenced by health status, family context, and other psychosocial factors not included in the present analysis, in addition to structural resources. Therefore, the findings of this study are more appropriately interpreted as revealing an overall association between structural resources and depression risk, rather than as providing a sufficient explanation of individual variation in CES-D scores.

### Elevated depressive symptoms: associations with survey-wave sample and structural resources

Table 3 presents the results of the binary logistic regression analysis using elevated depressive symptoms (CES-D ≥ 36) as the outcome. After adjusting for maternal age, marital status, self-rated health, and number of children, mothers in the 2022 sample had significantly higher odds of belonging to the elevated depressive symptoms group than those in the 2016 sample (OR = 2.56, 95% CI: 1.33–4.92, *p* = 0.005). Although mothers in 2018 and 2020 also showed a tendency toward higher risk, these associations did not reach statistical significance.

**Table 3:** Binary logistic regression analysis of elevated depressive symptoms among mothers, 2016–2022.

Variable	OR	95% CI	*p*
Survey year (ref: 2016)			
2018	1.36	0.82–2.24	0.233
2020	1.52	0.84–2.73	0.168
2022	2.56	1.33–4.92	0.005
Structural resource index	0.88	0.77–1.00	0.046
Maternal age	0.97	0.93–1.02	0.191
Marital status (married = 1)	0.91	0.46–1.79	0.786
Self-rated health	1.89	1.54–2.32	<0.001
Number of children	1.05	0.90–1.23	0.524

*n* = 588. OR indicates odds ratio, and CI indicates confidence interval. The model adjusted for maternal age, marital status, self-rated health, and number of children.

The structural resource index was negatively associated with elevated depressive symptoms. For each one-unit increase in the structural resource index, the odds of belonging to the elevated depressive symptoms group decreased by approximately 12% (OR = 0.88, 95% CI: 0.77–1.00, *p* = 0.046). However, given the borderline statistical significance of this result, the effect size and practical relevance should be interpreted cautiously.

Self-rated health was significantly and positively associated with elevated depressive symptoms (OR = 1.89, 95% CI: 1.54–2.32, *p* < 0.001), making it one of the most stable correlates in the model. In contrast, maternal age, marital status, and number of children did not reach statistical significance in the fully adjusted model.

Overall, compared with its association with continuous CES-D scores, the association between structural resources and elevated depressive symptom risk was more evident in the binary outcome model. This suggests that structural resources may be more closely reflected in the distinction of elevated-risk status than in the explanation of individual variation in continuous CES-D scores.

### Sensitivity analyses

#### Sensitivity analysis 1: sensitivity analysis using different CES-D thresholds

To examine the robustness of the findings, this study further used CES-D ≥ 40 as a stricter threshold for elevated depressive symptoms and repeated the logistic regression analysis. This threshold approximately corresponds to the conventional CES-D ≥ 20 criterion.

The results showed that, in the fully adjusted model, mothers in the 2022 sample still had significantly higher odds of elevated depressive symptoms than those in the 2016 sample (OR = 2.86, 95% CI: 1.32–6.16, *p* = 0.008).

In contrast, the association between the structural resource index and elevated depressive symptoms was no longer statistically significant under the stricter threshold (OR = 0.94, 95% CI: 0.77–1.14, *p* = 0.523). Self-rated health remained significantly and positively associated with elevated depressive symptoms (OR = 1.77, 95% CI: 1.37–2.27, *p* < 0.001).

Overall, the sensitivity analysis suggests that there may be an association between structural resources and elevated depressive symptoms, but this association was attenuated under the stricter depressive symptom threshold.

#### Sensitivity analysis 2: sensitivity analysis using the PCA-derived structural resource index

To further examine the robustness of the structural resource index construction, principal component analysis (PCA) was used to reduce the three indicators—educational attainment, employment status, and urban–rural residence—into an alternative composite measure.

The results showed that the first principal component explained approximately 50.6% of the total variance and was highly correlated with the original equal-weighted structural resource index (*r* = 0.989).

After using the PCA-derived structural resource index, the regression results were generally consistent with the main analysis. In the logistic regression model for elevated depressive symptoms (CES-D ≥ 36), the PCA-derived structural resource index showed a marginally significant negative association with elevated depressive symptoms (OR ≈ 0.86, *p* = 0.062). In the continuous CES-D outcome model, this association did not reach statistical significance.

Overall, the PCA sensitivity analysis was broadly consistent with the direction of the main findings, suggesting that the main results showed a certain degree of robustness across different methods of constructing the structural resource index.

## Discussion

Using four waves of data from the China Family Panel Studies (CFPS) from 2016 to 2022, this study examined between-sample differences in depressive symptoms among postpartum women across survey waves and assessed the association between structural resources and elevated depressive symptoms. The findings can be summarized in three main points. First, depressive symptom levels and the proportion of elevated depressive symptoms differed across survey-wave samples, with the highest proportion observed in the 2022 sample. Second, the structural resource index, which reflects socioeconomic position through educational attainment, employment status, and urban–rural residence, was negatively associated with elevated depressive symptoms. However, the overall effect size was modest, and the statistical significance of this association varied across model specifications and sensitivity analyses. Third, the association between structural resources and elevated depressive symptoms was more evident in the binary outcome model than in the continuous CES-D model. Overall, the findings suggest that differences in structural resources may be associated with postpartum mental health risk, particularly in distinguishing a relatively high-risk subgroup. Because this study used a repeated cross-sectional design, these findings should be interpreted as between-sample differences across survey waves rather than as longitudinal changes in postpartum depressive symptoms among the same individuals.

The negative association observed in this study between socioeconomic position and postpartum depression is generally consistent with a substantial body of international and Chinese research published in recent years. Existing studies have shown that women with lower educational attainment, unstable employment, or residence in settings with relatively limited social resources may be more likely to experience mental health problems during the perinatal or postpartum period (19–21). However, compared with some studies reporting stronger socioeconomic effects, the statistical association of the structural resource index in the present study was relatively limited. It was mainly observed in the binary model for elevated depressive symptoms, while it did not reach statistical significance in the continuous CES-D outcome model. This finding suggests that the relationship between structural resources and postpartum mental health may be complex and may not be fully captured by a single linear model. In addition, the structural resource index constructed in this study was based only on educational attainment, employment status, and urban–rural residence. It therefore mainly reflects mothers’ relative resource position within the social structure and does not cover other structural factors such as household income, housing conditions, access to social welfare, or community support environments. Accordingly, the operational definition of “structural resources” in this study still has certain limitations, and its theoretical scope should be interpreted cautiously. Nevertheless, the PCA sensitivity analysis produced results broadly consistent with the main analysis, suggesting that the direction of the findings was relatively robust across different methods of index construction. Therefore, the present findings provide preliminary evidence that composite resource conditions may help identify differences in postpartum mental health risk, although the measurement of structural resources in this study remains limited.

In the Chinese context, previous studies based on CFPS or other large-scale survey data have reported associations between socioeconomic inequality and mental health outcomes (22, 23). Therefore, the contribution of the present study does not lie in claiming that socioeconomic factors are newly identified predictors of postpartum depression. Rather, it lies in using repeated cross-sectional CFPS data to examine whether postpartum mothers’ structural resource position is associated with depressive symptom differences across survey-wave samples. Specifically, this study does not treat education, employment, and urban–rural residence merely as background control variables, but conceptualizes them as a composite indicator of mothers’ structural resource position. This approach highlights the potential relevance of resource distribution differences for postpartum mental health. At the same time, the results should be interpreted cautiously: the statistical effect was modest, and its significance varied across model specifications and sensitivity analyses. Thus, the findings are better understood as exploratory evidence of an association between structural resource position and elevated depressive symptom risk, rather than as direct verification of a structural mechanism.

Unlike many studies that focus primarily on perceived social support or family functioning as psychosocial constructs (24, 25), this study emphasizes structural resource conditions as a distinct analytical dimension. This does not imply that family support or caregiving arrangements are unimportant. Rather, the study highlights that different forms of support may operate at different levels. Emotional and instrumental support are often experienced within family interactions, whereas education, employment, and urban–rural residence reflect more durable social positions and resource conditions. In this study, the structural resource index showed a modest negative association with elevated depressive symptoms, although it was not significantly associated with continuous CES-D scores. This finding suggests that structural resource conditions may be more relevant to identifying relatively high-risk groups than to explaining the full range of individual variation in depressive symptom levels. Future studies with richer measures of family relationships, support quality, and caregiving arrangements are needed to further examine how structural resources and family support jointly shape postpartum mental health. Existing studies have often explained postpartum depression in terms of the presence of emotional or instrumental support, while paying relatively less attention to the social structural conditions in which support is embedded (26). Therefore, the present findings suggest, to some extent, that differences in long-term resource conditions may be associated with whether some postpartum women enter a relatively high-risk psychological state, compared with short-term and situational support arrangements. However, this finding requires further validation using richer measures of family relationships, quality of social support, and longitudinal follow-up data.

In recent years, research on grandparental childcare, intergenerational family support, and women’s mental health has increased substantially, yet the findings remain mixed. Some studies suggest that grandparental involvement in childcare may relieve mothers’ caregiving burden and reduce depression risk (27), whereas others suggest that intergenerational support may also be associated with relational strain or less favorable psychological outcomes in certain family contexts (28, 29). Taken together, the existing literature suggests that family caregiving support is not a universally protective factor with a single, uniform effect. Its mental health implications may depend on the specific form of support, the quality of interaction, and the family relationship structure in which it is embedded. This also suggests that family support should not be treated as a homogeneous protective resource; its actual role may be shaped by household resource conditions, gendered divisions of labor, and intergenerational relationship patterns.

The present findings should therefore be interpreted as highlighting the need to consider family support within broader structural conditions. The same caregiving arrangement may have different psychological implications depending on household resource conditions, intergenerational relationship patterns, and the distribution of childrearing responsibilities. Accordingly, understanding the relationship between family support and postpartum depression should move beyond the simple question of whether support exists and further consider how support is provided, who provides it, and under what structural conditions it operates. From this perspective, family support and structural resources should not be regarded as completely independent domains; rather, they may be mutually embedded in shaping postpartum mental health. However, because the present study did not include detailed measures of support quality, caregiving intensity, or family interaction processes, this interpretation should be regarded as a theoretical implication rather than direct empirical verification.

The 2022 survey-wave sample showed higher odds of elevated depressive symptoms than the 2016 sample after adjustment for available covariates. Given the repeated cross-sectional design, this finding should be interpreted as a between-sample difference rather than as evidence of worsening mental health among the same individuals over time. The 2022 difference may reflect, at least in part, broader social and family-contextual changes during this period. Previous studies have discussed the potential impact of the COVID-19 pandemic, labor market instability, and increased family caregiving pressure on women’s mental health (30–33). However, the present study did not include individual-level measures of pandemic exposure, income change, employment disruption, or care interruption. Therefore, no direct causal attribution can be made. The discussion of the broader social context should be understood as a cautious interpretation of time-varying background conditions rather than as evidence that any specific event caused the observed between-sample difference.

From a methodological perspective, this study did not employ complex causal inference methods or structural equation modeling. Its strengths instead lie in the use of nationally representative data, consistent measurement across multiple waves, and a relatively explicit logic for variable construction. In current research on postpartum mental health, much evidence still comes from single-region hospital samples, community samples, or small-scale cross-sectional surveys. Therefore, comparative analysis across different survey-year samples based on nationally representative survey data can still provide useful population-level evidence on differences in postpartum maternal mental health in China (34). In addition, by explicitly distinguishing different types of support-related variables, this study provides a clearer theoretical starting point for future research using more complex methods, such as mediation analysis, family-level models, or quasi-experimental designs. In particular, placing structural resources and family support within the same analytical framework helps facilitate further discussion of the possible links between social resource conditions and family support processes, rather than treating the two as entirely independent influences.

Nevertheless, the statistical models in this study still have several limitations. Although marital status, self-rated health, and number of children were included as covariates, unobserved confounding may remain. For example, marital quality, life events, parenting stress, postpartum physical recovery, and prior mental health history may all have important influences on postpartum depressive symptoms. At the same time, the income variable could not be included in the main models because of its high proportion of missing values, which also indicates that the measurement of structural resources in this study remains limited. Therefore, the explanatory power of the continuous CES-D outcome model was relatively limited, suggesting that individual variation in postpartum depressive symptoms may be jointly influenced by multiple psychosocial and health-related factors. Based on this, the findings of this study are more appropriately understood as exploratory evidence regarding the overall association between structural resources and elevated depressive symptom risk, rather than as a sufficient explanation of the mechanisms underlying postpartum depression or as strict causal inference. Based on these limitations, the findings of this study are more appropriately understood as exploratory evidence regarding the association between structural resources and elevated depressive symptom risk across survey-wave samples, rather than as a sufficient explanation of the mechanisms underlying postpartum depression or as evidence of causal change over time.

## Implications for social work practice: from family support to structural empowerment

The findings of this study provide empirical support for considering a more systematic role for social work in the prevention and intervention of postpartum depression. The results suggest that, compared with caregiving arrangements alone, structural resource conditions may show a more stable association with postpartum depression risk. This indicates that differences in postpartum depression risk may be related not only to whether caregiving support is available within the family, but also to mothers’ educational attainment, employment conditions, and access to broader social resources. Therefore, when understanding postpartum mental health, it is necessary to consider not only individual emotional states and family interactions, but also the broader social structural environment in which mothers are embedded.

From the perspective of the person-in-environment framework, postpartum depression can be understood as a manifestation of the imbalance between the social structural pressures women face during the transition to motherhood and the family role expectations placed upon them. In this process, reliance solely on caregiving assistance from family members may not be sufficient to alleviate the persistent pressures associated with limited educational opportunities, employment interruption, and insufficient access to social resources. By contrast, medical social workers may be able to assess mothers’ broader social environments, identify potential barriers related to social protection, employment support, family role negotiation, and access to community resources, and provide more targeted support on that basis. However, it should be noted that this study did not directly evaluate the effects of social work interventions. Therefore, the discussion of possible social work mechanisms should be understood as a practice-oriented inference based on the study findings rather than as direct empirical verification.

From the perspective of the stress–resource model and empowerment theory, the potential value of social work lies in transforming fragmented and short-term supportive behaviors into sustainable social resources that mothers can mobilize over time. Through case management, family negotiation, resource linkage, and policy advocacy, social work interventions may not only help relieve immediate stress but may also, to some extent, strengthen mothers’ decision-making capacity and room for action within both family and society. In light of the present findings, this approach may be particularly worth further exploration among mothers with relatively limited structural resources and unstable support networks. Nevertheless, given that the statistical association between structural resources and elevated depressive symptoms in this study was generally modest, these practice implications should be interpreted cautiously and require further validation in future research.

Accordingly, the findings of this study suggest that the prevention and intervention of postpartum depression should not be confined to caregiving arrangements within the household. Greater attention should also be paid to the integration of structural resources, community support services, and pathways of social empowerment. From the perspective of integrating public health and social services, maternal and child health systems may further explore the possibility of incorporating risk identification, resource linkage, family support coordination, and community referral mechanisms into routine maternal and child health services, in order to respond more effectively to social inequalities related to postpartum mental health.

This study has several limitations. First, the CFPS was not specifically designed for postpartum populations, and some key measures of psychological characteristics and relationship quality were relatively limited. Second, the childcare support variables could not fully distinguish differences in caregiving intensity, interaction quality, or sources of support. Third, the repeated cross-sectional design allows the identification of overall differences across survey waves, but does not support strict causal inference. Fourth, the final analytic sample sizes differed across waves, with the 2022 sample being relatively small, which may affect the statistical stability of year comparisons. Fifth, the structural resource index constructed in this study was a theory-driven equal-weighted composite indicator. Although this approach helps capture mothers’ overall resource conditions, it remains limited in identifying the relative contribution of different components. In addition, because the income variable had a relatively high proportion of missing values, it was not included in the main models. Therefore, the current findings remain limited in characterizing socioeconomic inequality. Furthermore, the definition of elevated depressive symptoms in this study was based on a relatively high-risk subgroup within the sample rather than on a clinical diagnostic threshold. The results are therefore more suitable for identifying risk differences than for estimating the clinical prevalence of postpartum depression.

Future research may be extended in several directions. First, more detailed variables on caregiving source, relationship quality, and support intensity could be introduced to examine the heterogeneous effects of different family support patterns. Second, longitudinal individual fixed-effects models or other stronger identification strategies could be used to reduce bias arising from unobserved heterogeneity. Third, postpartum depression could be examined within a broader framework of gender inequality and social policy (35). Fourth, future studies could be linked more closely to social work practice in community or healthcare settings to further test the mechanisms connecting improvements in structural resources, coordination of family support, and mental health outcomes. In addition, future research may further explore the cumulative effects, interactions, and dynamic changes among different dimensions of structural resources, in order to provide a more comprehensive understanding of the social determinants of postpartum mental health.

## Data Availability

Publicly available datasets were analyzed in this study. This data can be found at: Repository name: China Family Panel Studies (CFPS) Data Center, Institute of Social Science Survey, Peking University Direct link to the data: https://cfpsdata.pku.edu.cn. CFPS public-use datasets are accessed through the CFPS Data Center by dataset wave rather than by a single accession number.
